# Supplementary material for: Cardiac adverse events associated with chloroquine and hydroxychloroquine exposure in 20 years of drug safety surveillance reports
Source: Sci Rep. 2020 Nov 5;10:19199. doi: 10.1038/s41598-020-76258-0 (PMC7644696; doi:10.1038/s41598-020-76258-0)
Supplement: Supplementary file 1 — Supplementary Information. [file 41598_2020_76258_MOESM1_ESM.pdf]

Cardiac adverse events associated with chloroquine and hydroxychloroquine exposure in 20 years of drug safety surveillance reports

Isaac V. Cohen[1], Tigran Makunts[2,3], Talar Moumedjian[2], Masara A. Issa[2], Ruben Abagyan[2*]

[1] Clinical Pharmacology and Therapeutics, University of California San Francisco, San Francisco, California, United States

[2]Skaggs School of Pharmacy and Pharmaceutical Sciences, University of California San Diego, La Jolla, California, United States

[3]Oak Ridge Institute of Science and Education, Center for Drug Evaluation and Research, United States Food and Drug Administration, Silver Spring, Maryland, United States

*Correspondence to RA: rabagyan@health.ucsd.edu

Supplementary information

Table S1. List of all Cardiac ADR Terms

Cardiac ADR Term List
accelerated_idioventricular_rhythm
acquired_cardiac_septal_defect
acute_coronary_syndrome
acute_endocarditis
acute_left_ventricular_failure
acute_myocardial_infarction
acute_right_ventricular_failure
adams_stokes_syndrome
age_indeterminate_myocardial_infarction
alveolar_arterial_oxygen_gradient_increased
angina_bullosa_haemorrhagica
angina_pectoris
angina_unstable
angiocardiogram
anterior_spinal_artery_syndrome
aortic_arteriosclerosis
arrhythmia
arrhythmia_supraventricular
arrhythmogenic_right_ventricular_dysplasia
atrial_enlargement
atrial_fibrillation
atrial_flutter

atrial_hypertrophy
atrial_rupture
atrial_septal_defect
atrial_septal_defect_acquired
atrial_tachycardia
atrial_thrombosis
atrioventricular_block
atrioventricular_block_complete
atrioventricular_block_first_degree
atrioventricular_block_second_degree
atrioventricular_block_third_degree
atrioventricular_dissociation
basilar_artery_aneurysm
basilar_artery_occlusion
basilar_artery_stenosis
basilar_artery_thrombosis
biopsy_artery
biopsy_artery_normal
biopsy_heart
biopsy_heart_abnormal
bleeding_varicose_vein
bradyarrhythmia
bradycardia
bradycardia_foetal
bundle_branch_block
bundle_branch_block_bilateral
bundle_branch_block_left
bundle_branch_block_right
cardiac_ablation
cardiac_amyloidosis
cardiac_aneurysm
cardiac_arrest
cardiac_arrest_neonatal
cardiac_assistance_device_user
cardiac_asthma
cardiac_cirrhosis
cardiac_death
cardiac_discomfort
cardiac_disorder
cardiac_dysfunction
cardiac_enzymes_increased

cardiac_failure
cardiac_failure_acute
cardiac_failure_chronic
cardiac_failure_congestive
cardiac_failure_high_output
cardiac_fibrillation
cardiac_flutter
cardiac_function_test_abnormal
cardiac_hypertrophy
cardiac_imaging_procedure_abnormal
cardiac_index_decreased
cardiac_monitoring
cardiac_monitoring_abnormal
cardiac_murmur
cardiac_murmur_functional
cardiac_output_decreased
cardiac_output_increased
cardiac_perforation
cardiac_pseudoaneurysm
cardiac_sarcoidosis
cardiac_septal_defect
cardiac_septal_hypertrophy
cardiac_steatosis
cardiac_stress_test_abnormal
cardiac_tamponade
cardiac_valve_abscess
cardiac_valve_disease
cardiac_valve_rupture
cardiac_valve_sclerosis
cardiac_valve_thickening
cardiac_valve_vegetation
cardiac_ventricular_disorder
cardiac_ventricular_thrombosis
cardio_respiratory_arrest
cardio_respiratory_distress
cardiogenic_shock
cardiomegaly
cardiometabolic_syndrome
cardiomyopathy
cardiomyopathy_acute
cardiomyopathy_alcoholic

cardiopulmonary_failure
cardiospasm
cardiotoxicity
cardiovascular_deconditioning
cardiovascular_disorder
cardiovascular_function_test_abnormal
cardiovascular_insufficiency
cardiovascular_symptom
cardioversion
carditis
chronic_left_ventricular_failure
complications_of_transplanted_heart
congenital_arterial_malformation
congenital_atrial_septal_defect
congenital_cardiovascular_anomaly
congenital_cerebrovascular_anomaly
congenital_coronary_artery_malformation
congenital_heart_valve_disorder
congenital_heart_valve_incompetence
congenital_mitral_valve_incompetence
congestive_cardiomyopathy
coronary_angioplasty
coronary_arterial_stent_insertion
coronary_artery_aneurysm
coronary_artery_atherosclerosis
coronary_artery_bypass
coronary_artery_dilatation
coronary_artery_disease
coronary_artery_dissection
coronary_artery_embolism
coronary_artery_insufficiency
coronary_artery_occlusion
coronary_artery_perforation
coronary_artery_reocclusion
coronary_artery_restenosis
coronary_artery_stenosis
coronary_artery_surgery
coronary_artery_thrombosis
coronary_bypass_stenosis
coronary_bypass_thrombosis
coronary_endarterectomy

coronary_no_reflow_phenomenon
coronary_ostial_stenosis
coronary_revascularisation
coronary_vein_stenosis
cutaneous_vasculitis
defect_conduction_intraventricular
degenerative_mitral_valve_disease
diabetic_cardiomyopathy
diastolic_dysfunction
dieulafoy_s_vascular_malformation
diffuse_vasculitis
dilatation_atrial
dilatation_ventricular
disseminated_intravascular_coagulation
ductus_arteriosus_premature_closure
ductus_arteriosus_stenosis_foetal
ecg_signs_of_myocardial_ischaemia
echocardiogram
echocardiogram_abnormal
electrocardiogram
electrocardiogram_abnormal
electrocardiogram_ambulatory_abnormal
electrocardiogram_change
electrocardiogram_p_pulmonale
electrocardiogram_p_wave_abnormal
electrocardiogram_pacemaker_spike
electrocardiogram_poor_r_wave_progression
electrocardiogram_pq_interval_prolonged
electrocardiogram_pr_prolongation
electrocardiogram_q_wave_abnormal
electrocardiogram_q_waves
electrocardiogram_qrs_complex_prolonged
electrocardiogram_qrs_complex_shortened
electrocardiogram_qt_corrected_interval_prolonged
electrocardiogram_qt_interval_abnormal
electrocardiogram_qt_prolonged
electrocardiogram_qt_shortened
electrocardiogram_repolarisation_abnormality
electrocardiogram_st_segment_abnormal
electrocardiogram_st_segment_depression
electrocardiogram_st_segment_elevation

electrocardiogram_st_t_change
electrocardiogram_st_t_segment_abnormal
electrocardiogram_st_t_segment_elevation
electrocardiogram_t_wave_abnormal
electrocardiogram_t_wave_amplitude_decreased
electrocardiogram_t_wave_amplitude_increased
electrocardiogram_t_wave_inversion
electrocardiogram_t_wave_peaked
endarterectomy
endocardial_disease
endocardial_fibroelastosis
endocardial_fibrosis
endocarditis
endocarditis_fibroplastica
endocarditis_noninfective
endocarditis_q_fever
exercise_electrocardiogram_abnormal
extrasystoles
femoral_arterial_stenosis
foetal_arrhythmia
foetal_cardiac_disorder
foetal_heart_rate_abnormal
foetal_heart_rate_deceleration
foetal_heart_rate_deceleration_abnormality
foetal_heart_rate_decreased
foetal_heart_rate_disorder
foetal_heart_rate_increased
haemorrhage_coronary_artery
haemorrhagic_arteriovenous_malformation
haemorrhagic_vasculitis
heart_alternation
heart_block_congenital
heart_disease_congenital
heart_injury
heart_rate
heart_rate_abnormal
heart_rate_decreased
heart_rate_increased
heart_rate_irregular
heart_sounds_abnormal
heart_transplant

heart_valve_calcification
heart_valve_incompetence
heart_valve_insufficiency
heart_valve_operation
heart_valve_replacement
heart_valve_stenosis
hyperkinetic_heart_syndrome
hypersensitivity_vasculitis
hypertensive_cardiomegaly
hypertensive_cardiomyopathy
hypertensive_heart_disease
hypertrophic_cardiomyopathy
hypertrophic_obstructive_cardiomyopathy
in_stent_coronary_artery_restenosis
increased_ventricular_afterload
increased_ventricular_preload
internal_carotid_artery_kinking
interventricular_septum_rupture
intracardiac_mass
intracardiac_pressure_increased
intracardiac_thrombus
intraventricular_haemorrhage
intraventricular_haemorrhage_neonatal
ischaemic_cardiomyopathy
left_atrial_dilatation
left_atrial_enlargement
left_atrial_hypertrophy
left_to_right_cardiac_shunt
left_ventricular_dilatation
left_ventricular_dysfunction
left_ventricular_end_diastolic_pressure_decreased
left_ventricular_end_diastolic_pressure_increased
left_ventricular_failure
left_ventricular_heave
left_ventricular_hypertrophy
lipomatous_hypertrophy_of_the_interatrial_septum
low_cardiac_output_syndrome
ludwig_angina
lupus_endocarditis
lupus_myocarditis
lupus_vasculitis

malignant_hypertensive_heart_disease
malignant_pericardial_neoplasm
mean_arterial_pressure_increased
microvascular_coronary_artery_disease
mitral_valve_calcification
mitral_valve_disease
mitral_valve_disease_mixed
mitral_valve_incompetence
mitral_valve_prolapse
mitral_valve_repair
mitral_valve_replacement
mitral_valve_sclerosis
mitral_valve_stenosis
multiple_cardiac_defects
myocardiac_abscess
myocardial_bridging
myocardial_calcification
myocardial_depression
myocardial_fibrosis
myocardial_haemorrhage
myocardial_hypoxia
myocardial_infarction
myocardial_ischaemia
myocardial_necrosis
myocardial_necrosis_marker
myocardial_necrosis_marker_increased
myocardial_oedema
myocardial_rupture
myocardial_stunning
myocarditis
myocarditis_bacterial
myocarditis_infectious
myocarditis_mycotic
myocarditis_post_infection
myocarditis_rheumatic
myocarditis_septic
myopericarditis
nodal_arrhythmia
oedema_due_to_cardiac_disease
palpitations
paroxysmal_arrhythmia

patent_ductus_arteriosus
pericardial_calcification
pericardial_cyst
pericardial_disease
pericardial_drainage
pericardial_effusion
pericardial_excision
pericardial_fibrosis
pericardial_haemorrhage
pericardial_mass
pericardial_neoplasm
pericardial_operation
pericardial_rub
pericarditis
pericarditis_adhesive
pericarditis_constrictive
pericarditis_infective
pericarditis_lupus
pericarditis_rheumatic
pericarditis_uraemic
periprocedural_myocardial_infarction
pleuropericarditis
post_procedural_myocardial_infarction
postinfarction_angina
postpartum_venous_thrombosis
postpericardiotomy_syndrome
postural_orthostatic_tachycardia_syndrome
prinzmetal_angina
prosthetic_cardiac_valve_thrombosis
purulent_pericarditis
restrictive_cardiomyopathy
rheumatic_heart_disease
rhythm_idioventricular
right_atrial_dilatation
right_atrial_hypertrophy
right_atrial_pressure_increased
right_ventricular_dilatation
right_ventricular_dysfunction
right_ventricular_ejection_fraction_decreased
right_ventricular_enlargement
right_ventricular_failure

right_ventricular_heave
right_ventricular_hypertrophy
right_ventricular_systolic_pressure_decreased
right_ventricular_systolic_pressure_increased
scan_myocardial_perfusion_abnormal
silent_myocardial_infarction
sinoatrial_block
sinus_arrhythmia
sinus_bradycardia
sinus_node_dysfunction
sinus_tachycardia
somatoform_disorder_cardiovascular
stress_cardiomyopathy
stress_echocardiogram_abnormal
subacute_endocarditis
subclavian_artery_occlusion
subclavian_artery_stenosis
subclavian_artery_thrombosis
subclavian_vein_occlusion
subclavian_vein_thrombosis
subendocardial_ischaemia
sudden_cardiac_death
supraventricular_extrasystoles
supraventricular_tachyarrhythmia
supraventricular_tachycardia
systolic_dysfunction
tachyarrhythmia
tachycardia
tachycardia_foetal
tachycardia_induced_cardiomyopathy
tachycardia_paroxysmal
temporal_arteritis
torsade_de_pointes
ventricular_arrhythmia
ventricular_asystole
ventricular_dysfunction
ventricular_dyskinesia
ventricular_dyssynchrony
ventricular_enlargement
ventricular_extrasystoles
ventricular_failure

ventricular_fibrillation
ventricular_flutter
ventricular_hyperkinesia
ventricular_hypertrophy
ventricular_hypokinesia
ventricular_hypoplasia
ventricular_septal_defect
ventricular_septal_defect_acquired
ventricular_tachyarrhythmia
ventricular_tachycardia
ventriculo_vascular_shunt
wolff_parkinson_white_syndrome

Table S2. List of all NSAID Terms

NSAID Term List
Ampiroxicam
Celecoxib
Chlortenoxicam
Dexibuprofen
Diclofenac
Diflunisal
Droxicam
Etodolac
Fenoprofen
Firocoxib
Flufenamic
Ibuprofen
Indomethacin
Isoxicam
Ketoprofen
Ketorolac
Lornoxicam
Loxoprofen
Meclofenamic
Mefanemic
Meloxicam
Nabumetone
Naproxen
Parecoxib
Phenylbutazone

Piroxicam
Rofecoxib
Sulindac
Tenoxicam
Tolfenamic
Tolmetin
Valdecoxib

Table S3. Sample Size for Subsample

	Subgroup	Controls	Chloroquine	Hydroxychloroquine
**Full Set:**	*RA*	625,541	1,055	59,078
	SLE	14,449	225	5,926
**Subset with Age and Sex Available:**	*RA*	454,309	791	41,099
	SLE	9,249	179	4,572

Figure S1. Most Common ADRs Observed in Each Cohort

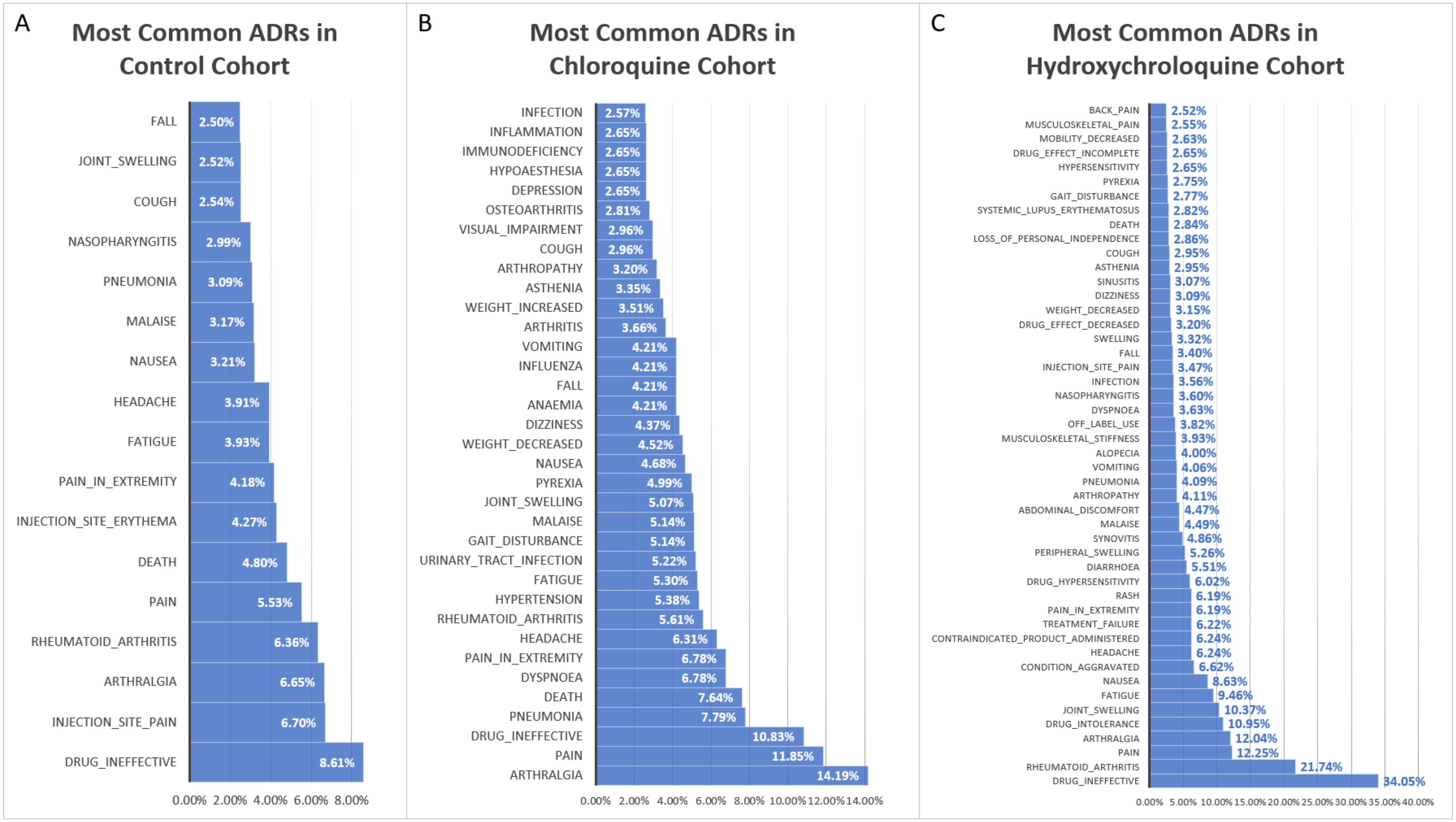

Figure S1 Legend. The most common adverse drug reactions (ADRs) with frequencies above 2.5% are presented for each of the three cohorts: (a) Control group, n = 639,990, (b) Chloroquine group, n = 1,280, and (c) Hydroxychloroquine group, n = 65,004.
